# Seeking a Role for Translational Control by Alternative Polyadenylation in *Saccharomyces cerevisiae*

**DOI:** 10.3390/microorganisms9091885

**Published:** 2021-09-05

**Authors:** Rachael E. Turner, Traude H. Beilharz

**Affiliations:** Development and Stem Cells Program, Monash Biomedicine Discovery Institute and Department of Biochemistry and Molecular Biology, Monash University, Melbourne, VIC 3800, Australia; rachael.turner@monash.edu

**Keywords:** alternative polyadenylation, 3′-end formation, *Saccharomyces cerevisiae*, translation

## Abstract

Alternative polyadenylation (APA) represents an important mechanism for regulating isoform-specific translation efficiency, stability, and localisation. Though some progress has been made in understanding its consequences in metazoans, the role of APA in the model organism *Saccharomyces* cerevisiae remains a relative mystery because, despite abundant studies on the translational state of mRNA, none differentiate mRNA isoforms’ alternative 3′-end. This review discusses the implications of alternative polyadenylation in *S. cerevisiae* using other organisms to draw inferences. Given the foundational role that research in this yeast has played in the discovery of the mechanisms of cleavage and polyadenylation and in the drivers of APA, it is surprising that such an inference is required. However, because advances in ribosome profiling are insensitive to APA, how it impacts translation is still unclear. To bridge the gap between widespread observed APA and the discovery of any functional consequence, we also provide a review of the experimental techniques used to uncover the functional importance of 3′ UTR isoforms on translation.

## 1. Introduction

Despite the close relationship between eukaryotic mRNA and protein expression, changes to mRNA concentrations do not always correlate to changes at the protein level. The 3′ untranslated region (3′ UTR) of mRNA plays a key role in regulating gene expression. In particular, mRNA stability, localisation and translation are largely impacted by the many *cis*-regulatory elements within the 3′ UTR. These interact with regulatory proteins that can negatively or positively influence the mRNA and its protein abundance. Therefore, changes to the length of the 3′ UTR have the potential to alter the fate of an mRNA.

In the budding yeast *Saccharomyces cerevisiae*, it is estimated that over 70% of genes contain multiple sites where cleavage and polyadenylation can occur [1]. mRNA isoforms produced from alternative poly(A) sites within the 3′ UTR generate the same protein; however, they differ in the length of their 3′ UTR. In such cases, the longer isoform tends to have a higher potential for regulation, due to additional regulatory elements present within the extended 3′ UTR. It is now clear from multiple gene-by-gene and transcriptome-wide studies that the relative concentration of the cleavage and polyadenylation factors, the rate of transcription, and chromatin architecture can influence poly(A)-choice [2,3,4,5,6]. However, whilst progress has been made towards understanding the effects of such alternative polyadenylation (APA) in mammalian systems [7], surprisingly little is known about its consequences in yeast.

In this review, we will describe what little is known about the effects of APA on translation in *S. cerevisiae* with inferences from other species. In addition, the different techniques available to better understand the impact that 3′ UTR isoform changes have on translation efficiency will be discussed.

## 2. The Regulatory Features Impacted by APA

**Figure 1:**
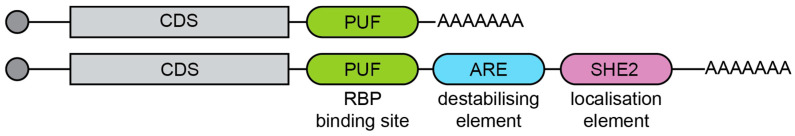
*Cis*-regulatory elements play a role in 3′ UTR isoform-specific translation. Alternative polyadenylation allows for the exclusion (top) or inclusion (bottom) of various *cis*-regulatory elements within the 3′ UTR. In yeast, these may include destabilising sequences such as A-rich elements (AREs), localisation elements such as She2p binding sites, and binding sites for *trans*-acting factors such as Pumilio family (Puf) proteins. Some *cis*-elements may be present in several isoforms. The sum effect of these different elements determines the overall stability, localisation, and translational efficiency of individual transcripts.

mRNA 3′ UTRs harbour many *cis*-regulatory elements that are involved in post-transcriptional regulation. Shortening or lengthening of the 3′ UTR thus results in the loss or gain of such regulatory sequences (Figure 1). In metazoans, these include microRNA (miRNA) binding sites. miRNAs are short, single-stranded RNAs of approximately 22 nt that base pair with target mRNA and cause destabilisation and translational repression of transcripts. Consequently, shorter mRNA isoforms tend to exhibit higher stability and increased protein levels due to the loss of miRNA target sites [8,9]. The long 3′ UTR isoform of the gene *Hip2*, for example, contains seed matches for the miRNAs *mir-21* and *mir-155* that are absent in the shorter isoform. Following T-cell activation, an increased relative expression of the shorter transcript compared to the longer isoform was accompanied by an increase in encoded ubiquitin conjugating enzyme levels [8]. Similarly, using luciferase reporter assays, mutation of the *miR-24* and *miR-155* seed matches within the longer 3′ UTR of *Hip2* restored luciferase expression to levels seen with the shorter 3′ UTR isoform. As such, shortening of the 3′ UTR tends to correspond with increased protein expression by eliminating miRNA-binding sites.

Mayr and Bartel [9] estimated that miRNA regulation evasion accounts for a quarter to two-thirds of the increase in protein expression levels related to shorter 3′ UTR transcript usage in human cancer cells. However, the impact that small RNA may have on translational control in fungi is less well understood. MicroRNAs, as well as small RNA (milRNA) and Dicer-independent Piwi-interacting RNAs (disiRNA), capable of gene repression through imperfect base-pairing, have been identified in the filamentous fungus *Neurospora crassa* [10]. However, neither these, nor miRNAs, have been found in *S. cerevisiae* and, consequently, antisense-mediated repression appears to have been lost in budding yeast [11]. As such, miRNA inclusion or exclusion does not alter the translatability of yeast mRNA. This eliminates a major contributor to APA-specific translation in yeast. Therefore, other regulatory events need to be taken into account. These include the effect of 3′ UTR length on nuclear export and translation, as well as changes to regulatory protein binding and cellular localisation caused by alternative 3′ UTRs.

### 2.1. Cis-Regulatory Features

The length of the mRNA itself has been seen to negatively influence the translation of a transcript. In yeast, shorter transcripts tend to form a more stable closed-loop structure, which enhances translation initiation and ribosome recycling [12]. However, shortening of the 3′ UTR may also negatively affect translational efficiency [13]. In mice, the transcription factor *BZW1* has three alternative 3′ UTR isoforms. Expression of these isoforms fused to *EGFP* indicated that the shortest transcript had the lowest protein expression [14]. This was thought to be linked to its short 3′ UTR length [15]. The length of the 3′ UTR has also been negatively linked to nuclear export rate in *Drosophila* [16]. This is likely a consequence of regulatory proteins binding to the 3′ UTR and slowing export or actively retaining mRNA transcripts within the nucleus. Such mechanisms are likely to occur also in yeast, where nuclear traffic is highly regulated [17].

Some studies have suggested that the presence of regulatory elements plays a larger role in 3′ UTR regulation than the length of the 3′ UTR itself [18]. Several sequence elements, such as AU-rich (AREs) and GU-rich (GREs) elements, have been associated with mRNA instability and decreased translation efficiency [19,20]. AREs occur in approximately 10% of human genes [21] and are conserved in yeast [22]. AREs tend to target mRNAs for rapid degradation in a deadenylation-dependent manner [19,23,24]. As such, ARE-containing mRNAs tend to be short-lived and generally encode proteins involved in cell growth or responses to external stimuli [25]. The loss of AREs by use of short 3′ UTR isoforms is likely to lead to increased mRNA stability and higher protein expression, relative to longer isoforms containing AREs.

Importantly, mRNA transcript stability and translational efficiency do not always correlate. The human gene for eukaryotic initiation factor 2α (EIF-2α) expresses both 1.6 kb and 4.2 kb mRNA products that differ in the length of their 3′ UTR sequence [26]. In activated T-cells, the longer 4.2 kb mRNA was more stable than the 1.6 kb mRNA. Despite both mRNA isoforms associating with polyribosomes, and, therefore, being actively and efficiently translated, the 1.6 kb mRNA produced two- to threefold more protein than the 4.2 kb mRNA in vitro. This suggests that the additional 3′ UTR sequence in the 4.2 kb isoform causes translation repression regardless of increased mRNA stability.

### 2.2. Trans-Regulatory Factors

*Trans* factors that interact with mRNA are also involved in alternative 3′ UTR isoform regulation. In *S. cerevisiae*, approximately 570 different proteins have been estimated to bind to RNA [27], and many proteins with other roles appear to moonlight as RNA-binding proteins [28,29,30,31]. These include proteins with the ability to stabilise or destabilise the transcript and regulate translational efficiency. Differential binding of such proteins between short and long isoforms is therefore likely to affect stability and/or translation. Surprisingly, no comprehensive analysis, mapping the intersect between APA and the binding sites of such RNA-binding proteins, has yet been attempted in yeast, despite the sequence motifs of a number of these being known [32].

Pumilio-Fem-3-binding factor (Puf) proteins are a conserved family of RNA-binding proteins that are linked to translational repression and have been associated with developmental regulation in several eukaryotes [33]. The first member of this family, Pumilio, is a *Drosophila* protein that binds to PUF response elements within the 3′ UTR of target mRNAs [34]. This binding promotes translational repression in both poly(A)-independent [35] and dependent manners [36,37]. One key role of Pumilio involves its binding to the maternal *hunchback* mRNA, causing its translational repression at the posterior pole during early embryogenesis [38,39,40,41].

In yeast, six different Puf proteins are known. Affinity-assay analysis of the Puf proteins 1–5 demonstrated interaction with approximately 12% of yeast mRNAs [42], and mutants lacking these Puf proteins caused a 7–8% difference in mRNA expression [43]. Interestingly, each Puf protein is associated with distinct groups of functionally related mRNA targets [42]. Puf1p and Puf2p bind to mRNAs of membrane-associated proteins, whereas Puf3p associates almost exclusively with mRNAs for nuclear-encoded mitochondrial proteins. In contrast, Puf4p and Puf5p interact preferentially with transcripts encoding proteins destined for the nucleus. Specifically, Puf4p binds to nucleolar ribosomal RNA-processing factor mRNAs, and Puf5p binds to mRNAs encoding chromatin modifiers and components of the spindle pole body. Differential inclusion of Puf binding sites within only longer alternative 3′ UTR mRNAs is likely to decrease protein production from these transcripts. Analysis of Puf binding sites and their association with different 3’ UTR isoforms would allow testing of APA-specific mRNA repression. However, to our knowledge, this analysis has not been systematically performed, even though, since both the consensus sequences for binding and the positions of APA are known, it would be bioinformatically straightforward to do so.

RNA-binding proteins can also increase the translational efficiency or stability of mRNA. HuR is a ubiquitously expressed mammalian RNA-binding protein that binds specifically to AREs within the mRNA transcript. This is generally associated with translocation to the cytoplasm and increased mRNA stability and translation [44,45,46,47]. Conversely, several instances of HuR promoting mRNA decay or repressing translation have also been reported [48,49]. Pub1p, a HuR-like protein, has been shown to similarly inhibit deadenylation and subsequent mRNA decapping in yeast [22]. Deletion of Pub1p, therefore, resulted in destabilisation of ARE-containing reporter transcripts. Similarly, the tristetraprolin family member, Cth2p, participates in the translational regulation and decay of AREs in targets, in a negative feedback loop of its own expression [50]. Again, whether such ARE-containing mRNAs are differentially regulated in conditions that stimulate APA is not known.

Sequences within the 3′ UTR have also been seen to affect the localisation of mRNA transcripts and their resulting proteins. The exclusion of such localisation sequences in shorter 3′ UTR isoforms is expected to alter expression within the cell. In neurons, two different transcripts of the brain-derived neurotrophic factor (*BDNF*) gene are produced that differ in the length of their 3′ UTR [51]. The shorter *BDNF* mRNA was found to be restricted to the cell body, whereas the longer isoform was preferentially targeted to dendrites [52]. The 3′ UTR sequence between the two *BDNF* poly(A) sites was also shown to be sufficient for targeting *GFP* mRNA to dendrites [52].

Currently, 32 mRNAs are known to specifically localise in yeast [53], though more are likely. Of these, 24 are targeted to the bud tip for localisation in the presumptive daughter cell [54,55,56,57,58]. This localisation is dependent on interaction with the RNA-binding protein She2p [54]. She2p then recruits the Myo4p–She3p complex that transports the mRNA to the bud tip [59,60,61,62]. The best characterised example is Ash1p, a protein involved in yeast cell-fate determination and inhibition of mating-type switching. The *ASH1* 3′ UTR is required for mRNA localisation to the distal tip of buds during anaphase of the cell cycle [56]. This results in the correct sorting of Ash1p to the nucleus of daughter cells. Consequently, 3′ UTR deletion caused the symmetrical distribution of Ash1p throughout the mother and daughter nuclei and prevented mating-type switching [56]. This was unable to be rescued when the 3′ UTR from *ADH2* was substituted [58]. However, the replacement of the *ASH1* 3′ UTR with that of *CDC6* caused only a 16% reduction in mRNA localisation and 10% reduction in Ash1p asymmetry.

This suggests that the *CDC6* 3′ UTR contains localisation signals, or that other parts of the *ASH1* mRNA play a role in targeting it to the bud tip [58]. Indeed, along with the discovery of a *cis*-regulatory element that overlaps with the termination codon and the 3′ UTR, three additional *cis*-elements have been located within the coding region of the *ASH1* mRNA [63]. These interact with She2p and are responsible for localisation to the presumptive daughter cell in a structure-specific, rather than sequence-specific, manner [58,59,61,63,64,65]. While each *cis*-element can individually target a reporter mRNA to the bud tip, having all four increased the quality and efficiency of *ASH1* mRNA localisation [65]. Shifting all four elements to the 3′ UTR did not affect mRNA localisation; however, it did affect the Ash1p asymmetry [65]. This was linked to the *cis*-elements reducing translation of *ASH1* mRNA during transport to daughter cells. In addition, the sixth yeast Puf protein, Puf6p, is also involved in *ASH1* mRNA translation repression and is required for its localisation through interaction with 3′ UTR Puf binding sites [66].

The other eight nuclear-encoded mRNAs known to specifically localise in yeast are targeted to the mitochondria in a 3′ UTR-dependent manner [67,68,69,70,71] and are believed to increase mitochondrial proteins’ import efficiency [69]. One such mRNA is *ATP2*. Substitution of the *ATP2* 3′ UTR with the 3′ UTR of non-localising *ADH1* inhibited mitochondrial targeting and caused respiratory dysfunction [69]. Therefore, 3′ UTRs play a key role in mRNA localisation in yeast, which is important for correct mitochondria biogenesis and bud tip asymmetry.

Additionally, 3′ UTRs are able to regulate protein localisation independently of mRNA localisation. Berkowitz and Mayr [72] demonstrated that alternative 3′ UTRs differentially regulate the localisation of membrane proteins post-translationally in human cell lines. The gene *CD47* possesses two alternative 3′ UTR isoforms. The long isoform enables cell surface expression, whilst the shorter transcript results in protein localised to the endoplasmic reticulum. This is a consequence of the longer 3′ UTR acting as a scaffold and recruiting HuR and SET proteins. During translation, this protein complex is targeted to the endoplasmic reticulum surface. The cytoplasmic domains of the CD47 protein can subsequently bind to SET, which interacts with Rac1 for translocation to the plasma membrane. Consequently, the 3′ UTR of mRNAs can play an integral role in mRNA and protein localisation. Shortening of this region through APA may therefore interfere with the subcellular localisation of mRNA and/or their resulting proteins. Whether such isoform-specific localisation mediated by APA occurs for yeast genes is still unknown.

## 3. Techniques for Investigation of Alternative 3′ UTR Isoform Translation

Given the widespread nature of alternative 3′ UTR usage and its potential functional consequences, further analysis of mRNA isoform-specific translation is of interest. Several key approaches currently exist for the examination of the translatome in yeast. These techniques will play a critical role in determining the influence that APA has on translation.

For individual genes, luciferase reporter assays are a common method for examining the effect of different 3′ UTR lengths and compositions on mRNA translational efficiency [8,9,73,74,75,76,77,78,79,80]. Different 3′ UTRs are fused to a luciferase reporter gene and the luciferase activity of different constructs is assessed. Decreased luminescence for one 3′ UTR length relative to another indicates a less translationally active isoform. Other useful reporter genes include GFP [52,81] and lacZ. However, some changes to translational efficiency between isoforms may be lost using this method. Fusion of alternative 3′ UTRs to a reporter gene is unlikely to demonstrate changes to the closed-loop structure formation, as the original gene’s length is not maintained and recapitulation of native 3’-end formation is complex. Moreover, the stability of these reporter proteins relative to the yeast cell cycle may mask subtle changes that have a more pronounced effect on short-lived proteins.

**Figure 2:**
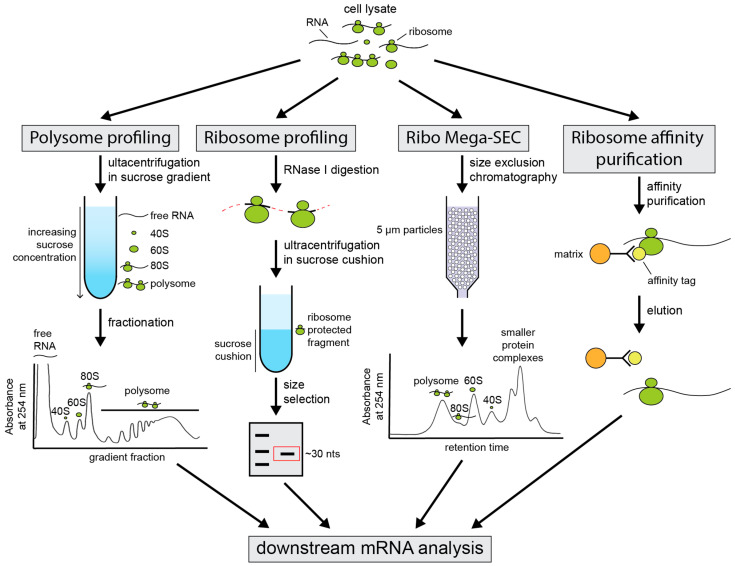
Experimental techniques to study the translatome. Cell lysate is prepared using the translation elongation inhibitor cycloheximide to stall ribosomes on mRNA transcripts. Polysome profiling separates cell lysate using ultracentrifugation in a sucrose density gradient that generally increases from 15% to 50% sucrose. Fractionation, whilst observing the absorbance at 254 nm, creates a polysome profile with peaks for free RNA, the 40S and 60S ribosomal subunits, the 80S monosome bound to RNA, and polysomes of increasing size. RNA is isolated from individual fractions for downstream applications such as qPCR, microarrays, or RNA-seq. Ribosomal profiling digests mRNA using RNase I. A translating ribosome encloses an approximately 30 nt sequence and protects it from nuclease digestion, creating ribosome-protected fragments (RPFs). Ribosomes are recovered by ultracentrifugation, usually in a sucrose cushion. RPFs are purified and size selected in a polyacrylamide gel for downstream applications. Ribo Mega-SEC cell lysate is injected into a uHPLC column containing 5 µm particles for separation, and a chromatogram is recorded during fractionation. This creates peaks for polysomes, monosomes, 60S and 40S ribosomal subunits, and smaller protein complexes or free RNA. RNA is isolated from individual fractions for downstream applications. Ribosome affinity purification uses affinity-tagged ribosomes to capture ribosomes and their associated mRNAs from cell lysate, using specific antibodies coupled to a matrix. Purified ribosomes are then released from the matrix and the mRNA prepared for downstream applications.

mRNA translational activity can also be inferred from its ribosomal association. Actively translated mRNAs will generally be bound to one or more ribosomes, known as monosomes and polysomes, respectively. Identification of an mRNA’s degree of ribosome association can, therefore, be used as a proxy for its translation efficiency. Several techniques have been designed that take advantage of this (Figure 2).

Polysome profiling remains the gold standard for the assessment of mRNA translational activity [82,83]. This technique uses sucrose-gradient centrifugation to separate actively translated polysome-associated mRNAs from untranslated free mRNA. Polysome profiling can be used to examine the translational efficiency of specific mRNAs gene-by-gene or, paired with microarray and deep-sequencing techniques, to assess translation transcriptome-wide [84,85,86,87,88]. As this approach retains the full-length mRNA following profiling, changes to the length of the 3′ UTR in translated and untranslated fractions and any ribosomal biases for specific isoforms can be monitored with 3′ UTR-specific sequencing methods. However, large amounts of starting material are required, and the procedure is labour intensive and not well-suited for handling many samples in parallel. Sucrose gradients also usually include heparin, which complicates the isolation of RNA for downstream applications. Furthermore, this conventional approach suffers from limited resolution and potential contamination of profiles with other high molecular weight complexes and membranes [89,90,91].

Resolution challenges were overcome with the development of the ribosome profiling technique [92] and its extension, TCP-seq [93]. Ribosomes leave an approximately 28–30 nucleotide footprint when bound to mRNA [92,94]. Following limited treatment with RNase I, only these ribosome-protected RNA fragments will remain. The libraries prepared from these fragments are then deep-sequenced to quantify ribosome positioning on individual genes globally to a sub-codon resolution [92]. However, this technique is not suitable for examining changes to translational efficiency linked to 3′ UTR APA. Despite providing higher resolution data, little information about non-coding regions, such as the 3′ UTR, is expected to be captured via this method. In yeast, 98.8% of ribosome footprints map to protein-coding regions of mRNA transcripts [92]. As such, ribosome profiling is blind to alternative 3′ UTR isoform usage and its effect on translation. Consequently, approaches that retain the entire mRNA transcript are preferable for analysis of 3′ UTR APA-dependent changes in translation efficiency.

An interesting variation on polysome profiling was recently developed to evaluate mRNA translational efficiency [95]. The Ribo Mega-SEC technique utilises size exclusion chromatography (SEC) and ultra high-pressure liquid chromatography (uHPLC) for the separation and analysis of polysomes and their associated mRNA molecules, rather than sucrose density gradients. This approach is expected to provide greater reproducibility than other methods, due to automated injection and fraction-collection systems [95]. Furthermore, unlike traditional polysome profiling, which includes a long ultracentrifugation step, Ribo Mega-SEC uHPLC runs for approximately 15 min. Optimisation of this approach would make it suitable for the analysis of translational efficiency in yeast.

An additional method for the evaluation of mRNA–ribosome association in yeast involves engineering ribosomes with an affinity tag. This allows rapid purification of ribosomes with their associated mRNA in vivo [96]. Ribosome affinity purification (RAP) uses genetically modified cells that express an affinity tag on a protein of the large ribosomal subunit. In yeast, these include the fusion of a FLAG(His)_6_ tag to Rpl25p [96] or incorporation of the protein A IgG-binding domain on Rpl16b [97]. Following affinity selection of ribosomes, their associated RNA is isolated for downstream applications such as RNA-seq. This provides a relatively quick and efficient method for translatome analysis using well-defined immunoprecipitation tags that can be adapted to high throughput assays. This technique is, however, lower resolution than polysome profiling, as the extent of polysome vs. monosome association cannot be quantified. Nevertheless, this remains a useful approach for determining whether a 3′ UTR isoform is translationally active.

Further refinement of these techniques or development of new translatome-focused methods would aid in accurate analysis of the translational efficiency in yeast. Careful consideration for the 3′ UTR is, however, needed to ensure new approaches do not obscure their role in translation. Three of the approaches—polysome profiling, Ribo-MEGA-Seq, and Ribosome affinity purification—are suitable for the determination of isoform-specific mRNA translation, yet they have not yet been utilised for this purpose, leaving the field open for new discoveries in translational control of alternative 3′ UTR isoforms.

## 4. Conclusions

*S. cerevisiae* represents a key model organism for basic biological research and has aided in a better understanding of human biology and disease. Though the majority of proteins that make up the yeast core cleavage and polyadenylation machinery have been identified, and much has been learnt about how alternative cleavage sites are selected [2], the role of this APA remains a relative mystery. The discovery of whether changes to the length of the 3′ UTR alter the translational efficiency of an mRNA transcript has been compromised by the techniques used to study the translatome. Consequently, as translational efficiency studies have progressed, the information present in 3′ UTRs has been left behind. The use of approaches allowing for analysis of the entire ribosome-associated mRNA or reporter assay systems will allow for further examination of the impact of APA on translation.

*S. cerevisiae* possesses fewer opportunities for APA-mediated control of translation due to the loss of miRNAs and their overall shorter 3′ UTR lengths (median 166 nucleotides [1]), compared to more complex eukaryotes. This model organism is also less likely to follow the “longer 3′ UTR transcripts create less protein” principle. This suggests a possibility that APA may exercise only a limited impact on translation in budding yeast. However, nutritional and pharmacological changes to cellular metabolism and transcription cause abundant changes to 3′ UTR lengths [2,3,4]. An option is that that this APA functions primarily as a transcription termination safety mechanism. However, various *cis*-elements present within the 3′ UTR and their associated regulatory proteins present a mechanism for 3′ UTR isoform-specific stability, localisation, and translation efficiency. Therefore, APA is likely to play a key role in translation in yeast. However, rather than global effects, the direction of control will likely be on a gene-specific basis, where translation is balanced by positive and negative *cis*-regulatory elements contained within the 3′ UTR isoform used. Research connecting mRNA translation to specific 3′ UTR isoforms is ripe for discovery after being overlooked for too long.
